# A novel xenonucleic acid-mediated molecular clamping technology for early colorectal cancer screening

**DOI:** 10.1371/journal.pone.0244332

**Published:** 2021-10-05

**Authors:** Qing Sun, Larry Pastor, Jinwei Du, Michael J. Powell, Aiguo Zhang, Walter Bodmer, Jianzhong Wu, Shu Zheng, Michael Y. Sha

**Affiliations:** 1 DiaCarta, Inc., Richmond, California, United States of America; 2 Weatherall Institute of Molecular Medicine, John Radcliffe Hospital, Oxford, United Kingdom; 3 Jiangsu Cancer Hospital & Jiangsu Institute of Cancer Research, Nanjing, China; 4 The Second Affiliated Hospital Zhejiang University, Hangzhou, China; University of Helsinki: Helsingin Yliopisto, FINLAND

## Abstract

**Background:**

Colorectal cancer (CRC) is one of the leading causes of cancer-related death. Early detection is critical to reduce CRC morbidity and mortality. In order to meet this need, we developed a molecular clamping assay called the ColoScape ^TM^ assay for early colorectal cancer diagnostics.

**Methods:**

Nineteen mutations in four genes (APC, KRAS, BRAF and CTNNB1) associated with early events in CRC pathogenesis are targeted in the ColoScape^TM^ assay. Xenonucleic Acid (XNA)-mediated qPCR clamping technology was applied to minimize the wild-type background amplification in order to improve assay sensitivity of CRC mutation detection. The assay analytical performance was verified and validated, cfDNA and FFPE CRC patient samples were evaluated, and an ROC curve was applied to evaluate its performance.

**Results:**

The data showed that the assay analytical sensitivity was 0.5% Variant Allele Frequency, corresponding to ~7–8 copies of mutant DNA with 5 ng total DNA input per test. This assay is highly reproducible with intra-assay CV of <3% and inter-assay CV of <5%. We have investigated 380 clinical samples including plasma cfDNA and FFPE samples from patients with precancerous and different stages of CRC. The preliminary assay clinical specificity and sensitivity for CRC cfDNA were: 100% (95% CI, 80.3–97.5%) and 92.2% (95% CI, 94.7–100%), respectively, with AUC of 0.96; 96% specificity (95% CI, 77.6–99.7%) and 92% sensitivity (95% CI, 86.1–95.6%) with AUC of 0.94 for CRC FFPE; 95% specificity (95% CI, 82.5%–99.1%) and 62.5% sensitivity (95% CI, 35.8%–83.7%) with AUC of 0.79 for precancerous lesions cfDNA.

**Conclusions:**

The XNA-mediated molecular clamping assay is a rapid, precise, and sensitive assay for the detection of precancerous lesions cfDNA and CRC cfDNA or FFPE samples.

## Background

Colorectal cancer (CRC) is one of the leading causes of cancer-related death and the third most common cancer with an estimated 1.8 million new cases worldwide in 2018 [1]. Colorectal cancer usually starts as a noncancerous growth called an adenoma or polyp that takes many years until it develops into cancer eventually [2, 3]. If detected at an early stage, CRC is treatable with a five-year survival rate of 90%, but if diagnosed at an advanced stage, the survival rate drops to 12–14% [4]. Early detection is critical to reduce CRC morbidity and mortality.

**Fig 1:**
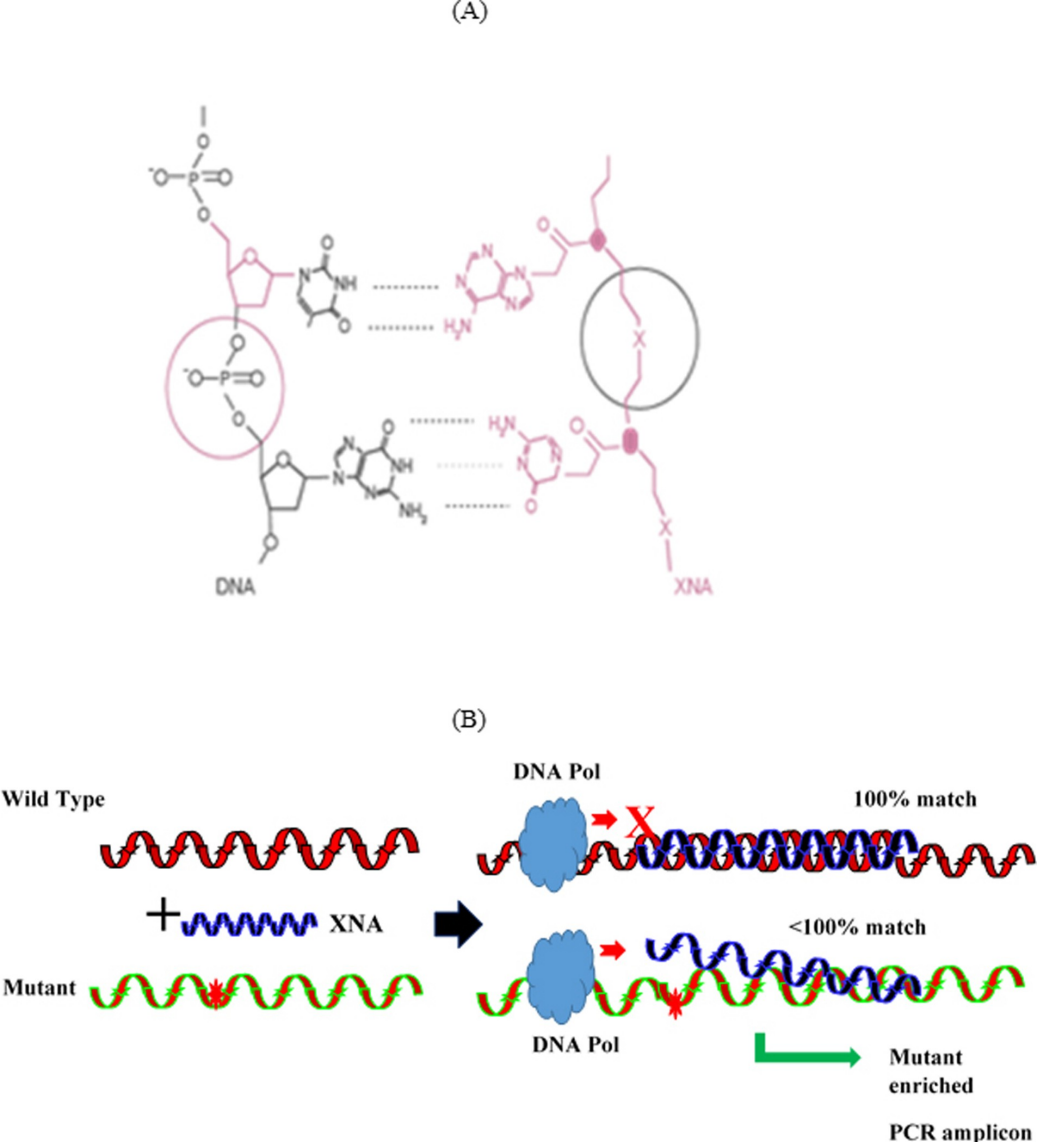
XNA structure and its function in the assay. (A). XNA structure and hybridization with DNA. (B). Principle of the Coloscape^TM^ assay mutation detection in targeted genes. XNAs hybridize tightly to complementary DNA target sequences only if the sequence is a complete match. When there is a mutation in the target site, and therefore is a mismatch, the XNA: DNA duplex is unstable, allowing strand elongation by DNA polymerase. Addition of an XNA whose sequence is a complete match to the wild-type DNA to a PCR reaction blocks amplification of wild-type DNA allowing selective amplification of mutant DNA. This enrichment of the mutation amplicons enables mutation detection by qPCR.

Several screening methods, including colonoscopy and guaiac-based fecal occult blood tests/fecal immunochemical tests (gFOBT/FIT) and FIT-DNA test, are recommended for CRC screening [2, 5–7]. Colonoscopy is still the standard method for CRC screening, but compliance with colonoscopy guidelines is low, possibly due to the invasive nature and the lengthy bowel preparation for the procedure, cost, and potential complications during the procedure (6). gFOBT/FIT tests have been widely used in CRC screening; however, the test sensitivity and specificity are low [8]. Sensitive and non-invasive methods for CRC screening are needed and advances in our understanding of molecular pathogenesis of CRC and molecular detection technologies now make this possible. Currently, non-invasive approaches include detection of genetic and epigenetic biomarkers associated with CRC in stool and plasma [9–11]. Complex signaling pathways are involved in colorectal cancer pathogenesis, including WNT and RAS /RAF/MAPK pathways, microsatellite instability (MSI, DNA mismatch repair) and some gene-specific CpG island methylation techniques [12–14]. Genetic and epigenetic changes in pathways have been studied extensively in relation to their roles in the initiation and development of CRC [15–28]. KRAS mutations occur in 36–40% of CRC patients with majority of mutations at codons 12, 13 and 61 [18, 22, 23]. The adenomatous polyposis coli (APC) gene is the key gene involved in the β-Catenin/Wnt signaling pathway and mutations in APC occur early and play an important role in colorectal tumorigenesis. The frequency of APC mutations ranges from 50% to 80% in CRC patients [13, 15–17]. BRAF is an oncogene that encodes a serine/threonine kinase that acts downstream of KRAS in the MAPK pathway [15, 26, 27]. BRAF mutations are present in about 10% of CRC with 90% of all BRAF mutations in CRC being BRAF V600E [27]. Molecular characterization of CRC and data on mutation incidence in CRC provided the basis for biomarker selection for our CRC mutation testing. A panel of target genes (APC, KRAS, BRAF, CTNNB1) was selected according to their mutation frequency in early-stage colorectal cancer [28–30]. One of the major challenges in cancer mutation detection is that clinical samples from cancer patients frequently contain trace amounts of mutant allele in a large excess of wild-type DNA, which hampers the sensitivity of mutation detection. Researchers have used different strategies such as Cast-PCR, Cold PCR, ARMS [31–33] and blocking oligonucleotides employed in PCRs (e.g. 3’ spacer, 3’ phosphate, 3’ ddC, etc.), as well as nucleotides with unnatural backbones such as peptide nucleic acid (PNA) and locked nucleic acid (LNA), to block or suppress wild-type effect on mutation detection [34, 35]. However, all the mutation detection assays employing the strategies and methods currently available still have limited sensitivity for detection of low-abundance variants, especially at early-stage cancer, when mutations are present in less than 1% variant allele frequency (VAF) or even much lower ratios of mutant to wild-type sequence. In this study, we applied the molecular clamping technology [36, 37] for minimally invasive and sensitive detection of CRC mutations from liquid biopsy or tumor tissue. This technology is employed to suppress amplification of wildtype alleles, thereby improving the sensitivity of mutation detection, especially for early adenomas and early-stage CRC. Xenonucleic acid (XNA) is a synthetic DNA analog in which the phosphodiester backbone has been replaced by a novel synthetically modified backbone chemistry (Fig 1A). XNAs are highly effective at hybridizing to targeted normal DNA sequences and can be employed as molecular clamps in quantitative real-time polymerase chain reactions (PCR) or as highly specific molecular probes for detection of nucleic acid target sequences [38] (Roberta D’Agata 2017). Binding of XNA to its target sequence blocks strand elongation by DNA polymerase in PCR assays. When there is a mutation in the target site, and therefore a mismatch, the XNA-DNA duplex is unstable, allowing strand elongation by DNA polymerase. Also, XNA oligomers are not recognized by DNA polymerases and cannot be utilized as primers in subsequent real-time PCR reactions (Fig 1B). Herein we report the development and validation of a novel XNA-based multiplex real-time PCR assay for the simultaneous and qualitative detection of somatic mutations in the genes frequently mutated in CRC patients. This multigene biomarker assay, called ColoScape^TM^, includes target gene mutation detection in APC, KRAS, BRAF, and CTNNB1 (Table 1).

**Table 1:** List of Coloscape^TM^ assay targeted gene mutations.

Genes	Exon	Amino Acid Change	Nucleotide change	Cosmic No.
KRAS	2	p.G12>A	c.35G>C	COSM522
		p.G12>R	c.34G>C	COSM518
		p.G12>D	c.35G>A	COSM521
		p.G12>C	c.34G>T	COSM516
		p.G12>S	c.34G>A	COSM517
		p.G12>V	c.35G>T	COSM520
		p.G13>D	c.38G>A	COSM532
		p.G13>C	c.37G>T	COSM527
		p.G13>R	c.37G>C	COSM529
APC	15	p.E1309fs*	c.3921_3925delAAAAG	COSM18764
		p.Q1367*	c.4099C>T	COSM13121
		p.R1450*	c.4348C>T	COSM13127
		p.R876*	c.2626C>T	COSM18852
CTNNB1	3	p.T41A	c.121A>G	COSM5664
		p.T41I	c. 122C>T	COSM5676
		p.S45P	c.133T>C	COSM5663
		p.S45F	c.134C>T	COSM5667
		p.S45del	c.133-135delTCT	COSM6128
BRAF	15	p.V600E	c.1799T>A	COSM476

## Materials and methods

### Reference materials and clinical samples

The following genomic DNA reference materials carrying specific mutations were obtained from ATCC and Horizon Discovery Group plc respectively: CTNNB1 S45 (HCT116), APC E1309 (LS1034), APC Q1367 (C2BBe1), APC R1450 (SW837), KRAS G12 (Horizon Cat#: HD272), KRAS G13 (Horizon Cat#: HD290), BRAF V600 (Horizon Cat#: HD238). For target mutations for which no commercial reference materials were available, APC, CTNNB1 synthetic DNA templates from Integrated DNA Technologies Inc were used. Reference cfDNA standards APC R1450, CTNNB1 T41, KRAS G12 and BRAF V600E were purchased from SeraCare Inc. For reference cfDNA standards that are not available commercially, genomic DNAs carrying APC E1309/Q1367, CTNNB1 S45 and KRAS G13 mutations were sheared by sonication with M220 Focused-ultrasonicator (Covaris Inc). The sonicated DNAs were analyzed on BioAnalyzer (Agilent) to give an average DNA fragment length of about 150 bp, which mimics the size of cfDNA fragments, justifying their use as cfDNA references.

Except for the 10 CRC whole blood samples were purchased from Discovery Life Sciences (Huntsville, AL, US), All FFPE and plasma (cfDNA) clinical samples with CRC used in this study were collected from Chinese patients (Second Affiliated Hospital of Zhejiang University, Hangzhou and Jiangsu Cancer Hospital, Nanjing, China). The ethics approval was awarded by the Ethics Committee of the Second Affiliated Hospital of Zhejiang University, Hangzhou, China. All subjects provided written informed consent. Samples were collected on Aug 2018-Aug 2019. 10 mL blood were drawn from each patient and stored in cfDNA BCT Streck tubes, following specific inclusion and exclusion criteria and sample handling guidelines for the patients. These samples were not specifically collected for this study, but were leftover samples from the lab, and the researchers were blinded from personal information associated with the samples.

### DNA extraction

DNA from FFPE samples was extracted with the QIAamp DSP DNA FFPE Tissue Kit (Catalog, Qiagen, REF 60604. QIAGEN GmbH, Hilden, Germany) following the manufacturer’s instructions. For cfDNA isolation, the collected blood was first spun at 1600xg on a table centrifuge (Sorvall ST16R, Thermo Fisher Scientific) for 10 minutes at room temperature. The supernatant above the interface phase was carefully taken and spun at 16,000xg for 10 minutes at room temperature. The final plasma supernatant was stored at –20°C until use. cfDNAs were isolated from plasma by using QIAamp ® MinElute ccfDNA Midi Kit (QIAGEN, Cat# 55284) following the manufacturer’s instructions. Isolated cfDNA was quantified by using Qubit dsDNA HS Assay Kit (Thermo Fisher Scientific, Cat # Q32851) and was also assessed by using the beta-actin qPCR assay (as internal control) to check the quantity and quality.

### qPCR primer and probe design

The high sensitivity of this multigene biomarker assay is achieved due to the XNA clamp probe technology. XNA oligomers that bind to the selected wild-type sequences at the respective genetic loci in the target genes were designed. For each of the selected mutation sites, primers and TaqMan hydrolysis probes were designed by Primer3 software version 0.4.0. For target gene codons with multiple mutations, (for example, KRAS G12 six mutations), a locus-specific probe was designed so that all the six KRAS G12 mutations could be detected in one assay using one pair of primers with the same XNA designed for the relevant position in KRAS G12. For target gene codons with single mutations, e.g., APC E1309, APC Q1367 and APC R1450 and APC R876, mutant-specific probes or allele-specific primers were designed. The human beta actin gene (ACTB) was selected as an internal control for the assay (S1 Table in S1 File). The designed primers and probes were analyzed in silico to verify the specificity of the oligos (by GenBank Blast against a whole genome reference DNA), absence of primer dimers (Auto-Dimer) and absence of amplicon secondary structure (M-fold) before synthesis. All primers were synthesized by IDT (Integrated DNA Technology) and probes were ordered from BioSearch Inc.

### XNA synthesis

XNA oligomers were synthesized in house or ordered from CPC Scientific Inc.

### ColoScape ^TM^ assay

The assay consisted of 10 μL of reaction volume, including 5 μL of 2X buffer (Bioline, Bio-11060), 2 μL of primer/probe mix in 1xTE with a final concentration of 100 nM-600 nM of primers and 50 nM– 500 nM of probes, 1 μL of XNA with final concentration from 0.125 μM to 1 μM and 2 μl of template (nuclease-free water for non-template control or 5–10 ng DNA). Non-template controls (NTC), negative controls (NC, human wildtype gDNA) and positive controls (PC, include each mutant DNA) were included in each run. The thermocycling profile was as follows: 95°C for 2 minutes followed by 50 cycles of 95°C for 20 seconds, 74°C for 40 seconds, 62°C for 30 seconds and 72°C for 30 seconds. The assay consisted of three multiplex qPCR reactions with XNAs to simultaneously detect all the indicated mutations (S2 Table in S1 File).

The mutational status of a sample was determined by calculating the Cq value between amplification reactions for a mutant allele assay and an internal control assay. Cq difference was determined as (ΔCq) = mutation assay Cq–internal control assay Cq. The cut-off values were experimentally determined as its ΔCq value by testing at least 20 wildtype gDNA and/or cfDNA repeatedly during the verification of assay performance. Cut-off ΔCq was calculated as ΔCq cut-off = ΔCq Cq average– 1.96*SD (at 99% CI). If the sample ΔCq was ≤ cut-off value, the mutation was detected as positive. If the sample ΔCq was > cut-off value, the mutation was not detected.

### Performance parameters of the assay

Performance parameters of the assay were established on DNA samples extracted from FFPE and plasma of CRC patients as well as reference materials. Assay performance characteristics were verified with respect to precision, limit of detection, specificity and cross-reactivity, as well as clinical sample validation and comparison with Sanger Sequence or NGS.

### Statistical analysis

We calculated the sensitivity, specificity, and Area Under Curve (AUC) of each group by sklearn.metrics [39]. ROC curves were then plotted by Matplotlib. Pyplot [40]. ROC curve and the area under the curve (AUC) were used to describe the assay performance. Positive Predictive Value (PPV), Negative Predictive Value (NPV) were calculated through Clinical Calculator 1 http://www.vassarstats.net/clin1.html

## Results

### Assay feasibility

**Fig 2:**
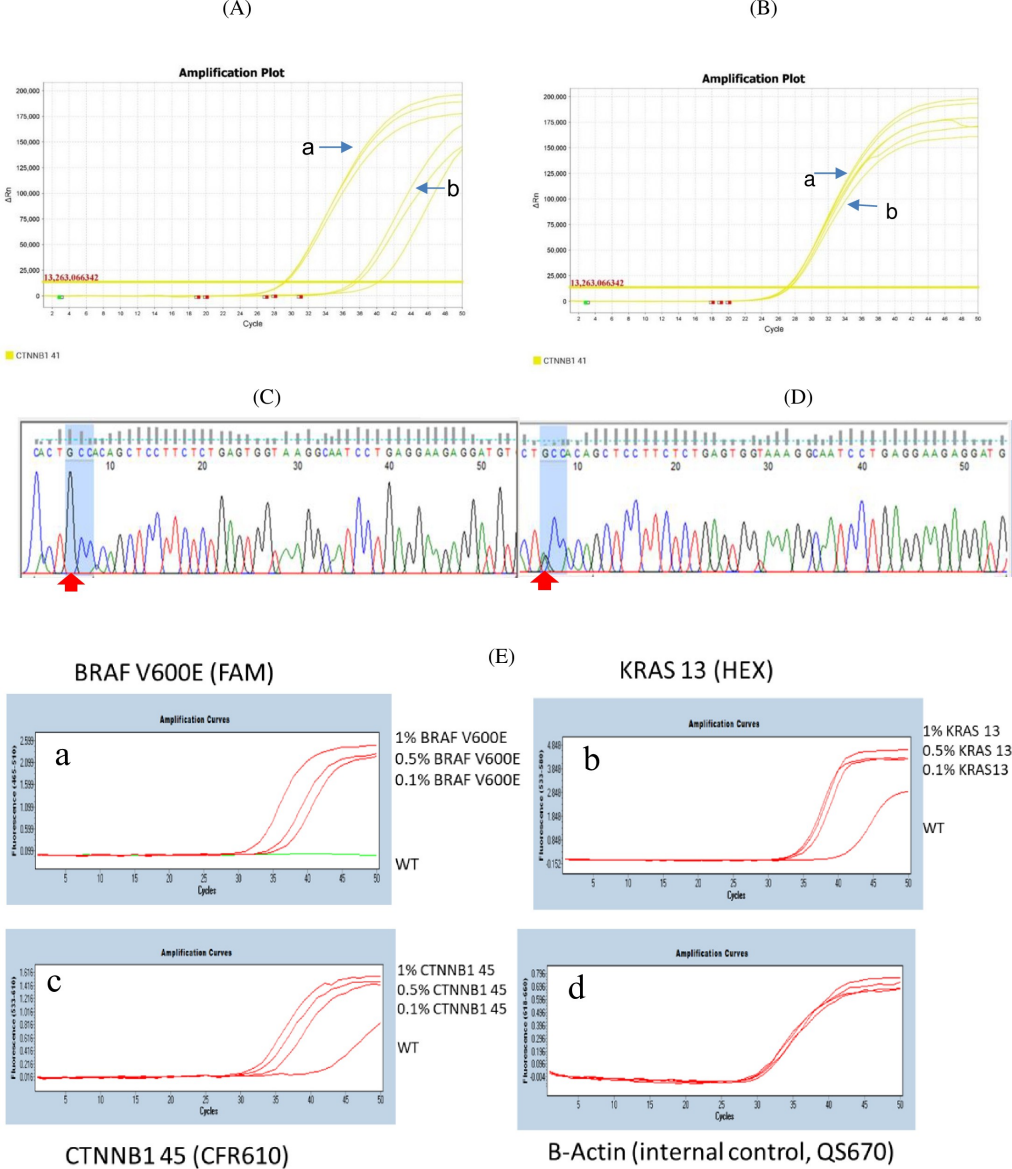
Comparison of XNA-based qPCR and qPCR without XNA. (A). CTNNB1 T41 amplification curves with XNA. a: 5% VAF of CTNNB1 T41 mutant, CT = 29.1 (ACTB, CT = 30.7, not shown here due to the use of Cy5 channel), b: CTNNB1 T41 wildtype, CT = 38.3 (ACTB, CT = 30). This indicates that XNA-based qPCR has a Δ Ct of ~9 for mutant to wildtype. (B). CTNNB1 T41 amplification curves without XNA. a, 5% VAF of CTNNB1 T41 mutant, CT = 26.9 (ACTB, CT = 30.4). b, CTNNB1 T41 wildtype, CT = 27.2 (ACTB, CT = 30). This indicates that qPCR without XNA has a Δ Ct of ~0.3 for mutant to wildtype. (C). Sanger sequencing for amplicons from CTNNB1 T41 assay with XNA, confirming that there is only mutant sequence GCC at CTNNB1 T41 (red arrow). (D). Sanger sequencing for amplicon from CTNNB1 T41 assay without XNA showing that there is a mix of wildtype and mutant A/GCC of CTNNB1 T41(red arrow). (E). Amplification profile of the ColoScape^TM^ multiplex qPCR assay with various concentrations of reference gDNA. a, BRAFV600E with 1%, 0.5%, 0.1% and 0% VAF (Fam as the probe-labeling dye). b. KRAS G13 with 1%, 0.5%, 0.1% and 0% VAF (Hex as the probe-labeling dye); c. CTNNB1 S45 with 1%, 0.5%, 0.1% and 0% VAF (CFR610 as the probe-labeling dye); and d. Beta-Actin (internal control, QS670 as the probe-labeling dye).

We designed the primers and probes for APC, CTNNB1, BRAF and KRAS, plus XNAs that cover 19 mutations of these four genes. To demonstrate that XNA can effectively suppress wild-type allele amplification and thus enrich the mutations detection during qPCR, we compared qPCR with and without XNA. The data show that the ΔCt was ~9 for mutant to wildtype for XNA-based qPCR (Fig 2A), while the ΔCt ~0.3 for mutant to wildtype for qPCR without XNA (Fig 2B). Sanger sequencing also confirmed that amplicons from XNA-based qPCR had a pure mutation reading (GCC for CTNNB1 T41A, Fig 2C) while amplicons from qPCR without XNA had a mixed reading of wildtype and mutation (A/GCC for CTNNB1 T41A, Fig 2D). XNA-based qPCR for other target gene mutants also showed the same pattern except for BRAF V600E (Fig 2E). This demonstrates that XNA enables mutation detection easily by blocking wildtype sequence amplification. For BRAF V600E, an allele-specific primer was designed to genotype BRAF V600E directly (Fig 2E_a). To verify that assay sensitivity was not compromised by multiplexing, we also compared singleplex and multiplex qPCR for each gene mutation in a 1% VAF reference DNA sample. The data showed that multiplex qPCR for each gene mutation had almost identical Cq value when compared to that of singleplex qPCR (S3 Table in S1 File).

### Assay analytical sensitivity

The analytical sensitivity of the ColoScape^TM^ assay was determined by studies involving APC, CTNNB1, KRAS and BRAF-defined genomic DNA reference samples. These known variant allele reference samples were diluted to 1% VAF, 0.5% VAF and 0.1% VAF separately. The reference samples were evaluated at 5 ng and 10 ng input. For all tested purified reference cfDNA, inputs were from 5–10 ng/well and all target mutations were detected with 100% correct calls at 0.5% VAF (Table 2), so the overall limit of detection (LOD) was 0.5% VAF. Moreover, sensitivity testing for APC Q1367, APC R1450, CTNNB1 T41, KRAS G12 &G13 and BRAF V600 in plasma even showed 0.1% VAF detection at 5 ng DNA (Table 2). For FFPE gDNA samples, the LOD for this ColoScape^TM^ assay was overall 0.5% VAF at 5 ng DNA input, which is about ~7–8 copies of mutant DNA (1 ng gDNA about 330 genomic copies). This was confirmed in three qPCR instruments ABIQS5, ABI 7500 FAST Dx and Roche LC480II (S4 Table in S1 File).

**Table 2:** Summary of assay limit of detection for the cfDNA sample.

Gene Target	Reference DNA input, ng/well		
	VAF%	10 ng cfDNA	5 ng cfDNA
		% Correct Call **	% Correct Call
APC E1309	1% VAF *	100%	100%
	0.5% VAF	100%	95%
	0.10% VAF	65%	40%
APC Q1367	1% VAF	100%	100%
	0.5% VAF	100%	100%
	0.10% VAF	100%	100%
APC R1450	1% VAF	100%	100%
	0.5% VAF	100%	100%
	0.10% VAF	90%	90%
CTNNB1 T41	1% VAF	100%	100%
	0.5% VAF	100%	100%
	0.10% VAF	100%	100%
CTNNB1 S45	1% VAF	100%	100%
	0.5% VAF	100%	100%
	0.10% VAF	90%	85%
KRAS G12	1% VAF	100%	100%
	0.5% VAF	100%	100%
	0.10% VAF	100%	95%
KRAS G13	1% VAF	100%	100%
	0.5% VAF	100%	100%
	0.10% VAF	90%	95%
BRAF V600	1% VAF	100%	100%
	0.5% VAF	100%	100%
	0.10% VAF	100%	95%

*VAF, Variant allele frequency. Reference standard materials were purchased from ATCC, Horizon or Seracare Life Sciences. The reference DNAs with defined VAF were diluted according to the assay requirement.

**Correct call: reference sample DNA with its defined VAF can be detected correctly.

### Assay precision

The high precision of the assay was verified by testing inter- and intra-assay, lot-to-lot (3 different lots), and operator and instrument variability. Instruments tested produced consistent results for the 1% mutant and wildtype gDNA controls. Ct values from three replicates were averaged and standard deviation and coefficient of variation (CV) were calculated. The precision studies indicate that this assay is highly reproducible with intra-assay CV of <3%, inter-assay CV of <5%, lot-to-lot CV of <4% and operator variability CV of <3%, indicating that the assay analytical precision is high.

### Assay analytical specificity

With the known reference wildtype gDNA, the assay specificity is over 97%. There were no false positive calls for up to 320 ng of gDNA per reaction and up to 20 ng FFPE DNA per reaction, with results confirmed by NGS. Overall, the analytical specificity of the assay was over 95% on reference gDNA and DNA extracted from FFPE or plasma.

Another aspect of assay specificity can be demonstrated by the evaluation of assay cross-reactivity. Each target assay was tested against all positive reference material to evaluate its cross-reactivity. All target mutations except KRAS G12 were detected as expected by the multiplex ColoScape^TM^ assay (Table 3), indicating that there is no cross-reactivity in these target detections. KRAS G12 produced a signal in KRAS G13 positive samples. However, there was a 6 Ct difference between the true KRAS G13 signal and the crosstalk signal from KRAS G12. Furthermore, since the kit is designed to detect KRAS G12 and KRAS G13 mutations separately in different tubes, the crosstalk will not have any impact on the assay performance. Therefore, only intended target mutations can be detected by the ColoScape^TM^ assay.

**Table 3:** Assay analytical specificity–the assay cross reactivity.

Assay Targets	Expected mutations in tested 5% VAF templates							
	APC E1309	APC Q1367	APC R1450	CTNNB1 T41	CTNNB1 S45	KRAS G12	KRAS G13	BRAF V600
APC E1309	**+**	**_**	**_**	**_**	**_**	**_**	**_**	**_**
APC Q1367	**_**	**+**	**_**	**_**	**_**	**_**	**_**	**_**
APC R1450	**_**	**_**	**+**	**_**	**_**	**_**	**_**	**_**
CTNNB1 T41	**_**	**_**	**_**	**+**	**_**	**_**	**_**	**_**
CTNNB1 S45	**_**	**_**	**_**	**_**	**+**	**_**	**_**	_
KRAS G12	**_**	**_**	**_**	**_**	**_**	**+**	*	**_**
KRAS G13	**_**	**_**	**_**	**_**	**_**	**_**	**+**	_
BRAF V600	**_**	**_**	**_**	**_**	**_**	**_**	**_**	**+**

+ Indicate mutant detection

- Indicate no cross reaction

*Indicates cross reaction

### Clinical performance

Clinical sensitivity and specificity were evaluated on FFPE and plasma of patients with different stages of CRC from normal to advanced adenomas (AA), to CRC stages I through IV. 380 patient samples, including 185 FFPE and 195 cfDNA samples, were tested during different experimental periods. In each case, a sample was considered positive if at least one of the target mutations tested positive. The test result was considered correct if the CRC samples and health samples were confirmed by Sanger sequencing or NGS (S5 Table in S1 File).

To compare the ColoScape ^TM^ assay and NGS, eighteen cfDNA samples of CRC patients were tested. The data showed that the ColoScape ^TM^ assay and NGS had a concordance rate of 89% (Table 4). There were two samples (DC04 and DC07) identified as mutants by the ColoScape™ assay and confirmed by Sanger sequencing, but not detectable by NGS.

**Table 4:** Comparison of the ColoScape ^TM^ assay and NGS for CRC cfDNA samples.

Patient ID	ColoScape™ Assay Results	NGS Results
DC01	Negative	Negative
DC02	KRAS G12 Positive	KRAS G12C
DC03	KRAS G12 Positive	KRAS G12D
DC04	KRAS G12 Positive	Negative
DC05	KRAS G12 Positive	KRAS G12C
DC06	APC E1309/1367 Positive, KRAS 12 Positive	APC E1309FS*
DC07	KRAS G12 Positive	Negative
DC08	KRAS G13 Positive	KRAS G13
DC09	KRAS G12 Positive	KRAS G12C
DC10	KRAS G12 Positive	KRAS G12
DC11	Negative	Negative
DC12	Negative	Negative
DC13	KRAS G12 Positive	KRAS G12D
DC14	APC R1450/876 Positive, KRAS G12 Positive	KRAS G12D
DC15	KRAS G13 Positive	KRAS G13D
DC16	KRAS G12 Positive	KRAS G12D
DC17	KRAS G12 Positive	KRAS G12C
DC18	KRAS G13 Positive	KRAS G13D

We also compared the ColoScape ^TM^ assay and Sanger sequencing (amplicon of qPCR) of 97 FFPE samples, which showed that the ColoScape ^TM^ assay had a 98% concordant rate with Sanger sequencing (S6 Table in S1 File).

To investigate whether plasma cfDNA and FFPE differ in CRC mutations detection from the same patient, we tested 11 pairs of matching tumor FFPE (containing some adjacent normal tissues) and cfDNA samples covering CRC stages I–IV. (Table 5). Interestingly, 100% cfDNA and 91% FFPE showed detectable mutations. The concordance for FFPE and cfDNA was about 90% (10/11 samples). Only one patient was identified with a different mutant for their cfDNA and FFPE paired samples (DCS9).

**Table 5:** Comparison of pairs of cfDNA and FFPE samples from the same CRC patient.

Patient ID	Diagnosis	cfDNA sample	FFPE sample
DCS01	Stage 4 Ca	KRAS G13	APC R1450 and KRAS G13 positive
DCS02	Stage 3b Ca	CTNNB1 S45, KRAS G13	KRAS G13
DCS03	Stage 3b Ca	BRAF V600 positive	BRAF V600 positive
DCS04	Stage 3b Ca	APC R1450 positive	APC R1450 positive
DCS05	Stage 1 Ca	KRAS G13	negative
DCS06	Stage 2 Ca	BRAF V600 positive	BRAF V600 positive
DCS07	Stage 2 Ca	CTNNB1 S45, BRAF V600 positive	BRAF V600 positive
DCS08	Stage 3 Ca	APC R1450 and BRAF V600 positive	BRAF V600 positive
DCS09	Stage 2a Ca	BRAF V600 positive	KRAS12 positive
DCS10	Stage 3b Ca	BRAF600 positive	KRAS G12 and BRAF V600 positive
DCS11	Stage 3b Ca	CTNNB1 S45 and KRAS G12 positive	KRAS G12 positive

*Each pair of cfDNA and FFPE came from the same patient.

**Fig 3:**
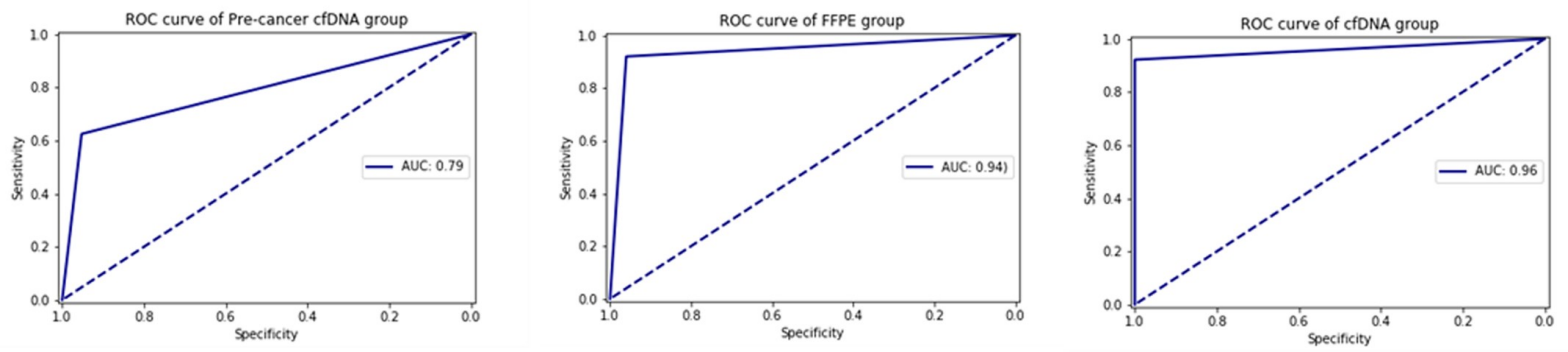
Receiver Operating Characteristic (ROC) curve and area under the curve (AUC) to evaluate the performance of the assay. a, precancerous lesions cfDNA samples analysis. b, CRC FFPE samples analysis; c, CRC cfDNA sample analysis.

For precancerous screening, we tested 10 advanced adenoma samples (AA, FFPE) and detected 6 true positives and 3 false negatives and confirmed its sensitivity at 66.6% (Table 6). For precancerous screening with cfDNA, 58 plasma samples from FIT + patients were tested using this assay and colonoscopy (S7 Table in S1 File). There were 2 false positives with 40 true negative samples, yielding a specificity of about 95.2%. There were 6 false negatives with 10 true positive samples that were confirmed by Sanger sequencing, corresponding to a sensitivity of about 62.5% for this cfDNA precancerous screening. This preliminary data indicates that this assay has a 62.5% –66% sensitivity for precancerous screening (AA FFPE 66.6% [95% CI, 30.9%–90.9%]; and cfDNA 62.5% [95% CI, 35.8%–83.7%]) and the specificity for precancerous cfDNA was 95.2% (95% CI, 82.5%–99.1%), with AUC of about 0.79 (Table 6 and Fig 3A). Excluding precancerous screening samples for CRC FFPE, the sensitivity was 92% (95% CI, 86.1%–95.6%) specificity was 96% (95% CI, 77.6%–99.7%), and AUC was about 0.94 (Table 6 and Fig 3B), while for CRC cfDNA, sensitivity was 92.2% (95% CI, 80.3%–97.5%), specificity was 100% (95% CI, 94.7%–100%) and AUC was 0.96 (Table 6 and Fig 3C). The assay accuracy was about 92.5% for CRC FFPE and 97% for CRC cfDNA separately (Table 6).

**Table 6:** Clinical sensitivity and specificity for FFPE and cfDNA samples.

Types of Clinical Samples	Patient number	Specificity	Sensitivity	Accuracy	PPV	NPV
CRC FFPE	175	96.0%	92.0%	92.5%	99.20%	66.60%
CRC cfDNA	137	100.0%	92.2%	97.0%	100%	95.50%
Advanced Precancerous lesions (AA *, FFPE)	10	NA	66.60%	66.6%	100%	NA
Precancerous lesions (cfDNA)	58	95.20%	62.50%	86.2%	83.30%	86.90%

* AA, Advanced Adenomas

## Discussion

In this study, we have demonstrated that the ColoScape ^TM^ assay is a robust CRC mutation detection assay with the analytical sensitivity (LOD) of about 0.5% VAF for cfDNA and FFPE. For some targets, the LOD was up to 0.1%VAF with preliminary clinical sensitivity about 92% and specificity about 96–100%; however, this needs to be followed up by a clinical trial for confirmation. The ColoScape ^TM^ assay is based on the XNA-mediated molecular clamping technology. XNA has a novel unique chemical backbone that is distinguished from the ‘classical’ PNA by the replacement of the backbone methylene functionalities by a heteroatom, which endows XNA molecular clamps with a high binding affinity for both DNA and RNA templates and higher melting temperature differentials in SNV and indels against natural DNA. The effectiveness of XNA in suppressing wild type amplification has also been demonstrated in mutation detection using a ‘classical’ PNA PCR clamp detection assay or by Sanger sequencing or NGS [34, 35]. XNA is thus confirmed to be a novel oligo blocker that has the potential to be applied to a variety of cancer mutation detection assays to improve assay sensitivity. Araki et al. reported that PNA-Clamp SmartAmp2, which is based on a principle similar to that of the ColoScape™ assay, can detect as little as 1% of the mutant allele of KRAS G12 in the DNA samples [41]. However, our assay can detect KRAS G12 even as low as 0.1% VAF from cfDNA samples (Table 2). Another advantage of XNA is that the mutations that are in the genomic region can be covered by XNA and detected. As shown in this study, only one pair of primers and probe are needed in the assay for the detection of all six genotypes of KRAS G12 mutations, which makes multiplexing of more target mutation detection easier due to the reduced need for PCR primers and probes and with reduced competition among reaction components. The LODs of different variants range from 0.2% to 2.5% VAF (S8 Table in S1 File), possibly due to variations in the nature of primer-template mismatches. This is consistent with the observations on the effects of primer-template mismatches on the detection and quantification of nucleic acids [42].

The preliminary clinical performance of the ColoScape ^TM^ assay showed good sensitivity, including the testing of precancerous screening samples. Mutations from early cancer patients were detected by the ColoScape ^TM^ assay and were confirmed by Sanger sequencing. The ColoScape™ assay and Sanger sequencing results were 98% concordant. The ColoScape™ assay has about 89% concordance with NGS (Table 4). There were two samples identified as mutants by the ColoScape™ assay and confirmed by Sanger sequencing, but not detectable by NGS, possibly due to the higher assay sensitivity (LOD about 0.1% VAF) of the ColoScape™ assay than that of NGS (LOD about 1% VAF). The ColoScape™ assay was used in testing precancerous plasma from FIT-positive patients in a small study and showed 83.3% positive predictive value (PPV) and 62.5% assay sensitivity, while FIT testing showed a PPV of 25%. Since FIT has such a high false negative rate, the ColoScape™ assay can potentially be used as a triage test in combination with a FIT test to improve the effectiveness of CRC patient management and treatment. There are several CRC-related mutation detection and methylation-based screening assays, (for example, Cologuard (Exact Sciences Corporation) and Epi ProColon® (Epigenomics AG)), available in the market. However, these FDA-approved assays are based only on a single gene methylation detection (Septin 9-Epi ProColon) or two methylation biomarkers combined with only one target gene mutation assay (Cologuard), which potentially limits the assay sensitivity. In this preliminary study, we have shown that the ColoScape ^TM^ assay can detect 66.6% of mutations from advanced adenoma (AA) samples (although it is only from 10 samples and needs further confirmation), while the reported Cologuard AA detection rate is 42–46% [43, 44]. This suggests that the ColoScape ^TM^ assay potentially has a comparable sensitivity to that of Cologuard for early colon cancer detection from patient blood samples. Furthermore, we have demonstrated a sensitivity for cfDNA sample for precancerous lesions of about 62.5% and specificity of 95.2%, suggesting that the ColoScape ^TM^ assay has great potential for early precancerous lesions screening.

## Conclusion

We have shown that the ColoScape ^TM^ assay has a robust analytical performance, and our preliminary data indicates clinical accuracy for FFPE and plasma samples (Tables 2 and 5 and S6 Table in S1 File). This rapid, precise, and sensitive molecular assay for mutation detection in CRC has the following key benefits: (a) The assay is unique. It covers a selection of multiple clinically relevant mutations in four genes and the proprietary XNA QClamp® TaqMan-based PCR technology has a high mutation detection sensitivity compared to other methods. (b). The assay is easy to use. Multiplex qPCR assay enables easy assay setup by end users. (c). The assay is efficient. Only 15–30 ng DNA is needed for assay input. (d). The assay is specific and sensitive. No cross-reactivity with wild-type is observed even with up to 320 ng purified gDNA. With 10 ng cfDNA or FFPE DNA per reaction, mutations can be detected at 0.5% VAF and even up to 0.1% VAF. (e) Preliminary assay clinical specificity is about 95–100% depending on the sample type (f). The assay reaction is rapid. Total run time is less than 3 hours. (g). The assay is versatile: assay is validated on widely used real-time qPCR machines. This open instrument system is more convenient for different clinical laboratories and end users.

In summary, we have developed a rapid and sensitive assay to enable the molecular characterization and detection of precancerous CRC and CRC at different stages in a variety of samples. However, this XNA-based qPCR with only five channels available in a qPCR instrument has its limitations. But the assay can be improved by targeting more genes and more mutation hotspots. Recent molecular characterization and NGS analysis of cancer patients has provided unprecedent insight into cancer molecular mechanisms and revealed molecular signatures of different cancer types. Inclusion of a broader biomarker panel will further improve the assay sensitivity. This XNA-based technology can also be combined with NGS technology to cover a wide range of variants in many genes, making it applicable to the development of comprehensive DNA-based assays for a wide range of cancer diagnostics.

## Supporting information

S1 File (DOCX)

## Abbreviations

XNA: Xenonucleic Acid
PNA: peptide nucleic acid
LNA: locked nucleic acid
CRC: colorectal cancer
gFOBT/FIT: guaiac-based fecal occult blood tests/fecal immunochemical tests
VAF: variant allele frequency
APC: adenomatous polyposis coli
BRAF: B-type Raf kinase
CTNNB1: Catenin beta-1
cfDNA: Circulating free DNA
